# A defined multi-metabolite formulation derived from Salvia miltiorrhiza alleviates obesity-associated cardiac lipotoxicity via modulating mitochondrial dynamics

**DOI:** 10.3389/fphar.2026.1674821

**Published:** 2026-04-30

**Authors:** Hetong Xu, Jing Kong, Ruoyu Wu, Zefei Chu, Weiya Zhang, Xiaoyi Zhao, Zhongxia Zhang, Shengjun An

**Affiliations:** 1 Hebei Provincial Engineering Laboratory of Plant Bioreactor Preparation Technology, Shijiazhuang, Hebei, China; 2 Department of Research Center, Hebei University of Chinese Medicine, Shijiazhuang, Hebei, China; 3 Hebei Key Laboratory of Healthcare with Traditional Chinese Medicine, Shijiazhuang, Hebei, China

**Keywords:** lipotoxic cardiomyopathy, mitochondrial dynamics, obesity, SABP, *Salvia miltiorrhiza*

## Abstract

**Background:**

Obesity is a major risk factor for lipotoxic cardiomyopathy, a process involving lipid metabolism disorders, inflammatory responses, and mitochondrial dysfunction. *Salvia miltiorrhiza (Danshen)*, a traditional Chinese medicinal plant, contains water-soluble metabolites with multitarget regulatory potential. Although some monomers of *S. miltiorrhiza* have been proved to be effective in cardiovascular diseases, the combined effects of metabolites and the mechanism remains unclear. This study aimed to investigate the cardioprotective effects and potential mechanisms of SABP, a defined formulation of four metabolites derived from *S. miltiorrhiza* (danshensu, salvianolic acid A, salvianolic acid B, and protocatechuic aldehyde), against lipotoxic myocardial injury, with a focus on the regulation of mitochondrial dynamics.

**Methods:**

A palmitic acid (PA)-induced lipotoxicity model in H9c2 cardiomyocytes and a high-fat diet (HFD)-induced obesity model in *ApoE* ^−/−^ mice were established. *In vitro*, the effects of SABP on cell viability (CCK-8), mitochondrial membrane potential (JC-1), mitochondrial dynamics-related proteins (Drp1, Fis1, Mfn2, Opa1), and apoptosis-related proteins (cleaved caspase-3, caspase-3) were assessed. *In vivo* evaluations involved fat distribution (Micro-CT), cardiac function (echocardiography), cardiac structure (HE, Masson staining, and TEM), serum lipid levels, inflammatory factors, cardiac enzyme activity (ELISA), and protein expression analysis in cardiac tissue (Western blot).

**Results:**

*In vitro* results showed that SABP significantly improved PA-induced reductions in cell viability and mitochondrial membrane potential, downregulated Drp1 and Fis1, upregulated Opa1 and Mfn2, and decreased the cleaved caspase-3/caspase-3 ratio. *In vivo*, SABP intervention reduced heart weight index and heart-tibia ratio, improved left ventricular systolic function, decreased fat accumulation and serum lipid abnormalities, attenuated inflammatory cytokine and myocardial enzyme levels, alleviated myocardial structural damage and fibrosis, and restored mitochondrial morphology. Western blot findings in cardiac tissue were consistent with *in vitro* results.

**Conclusion:**

SABP may protect against cardiac lipotoxic injury by improving lipid metabolism, reducing inflammation, and restoring mitochondrial dynamics homeostasis, highlighting its potential as a multi-component herbal therapy for metabolic cardiomyopathy and providing experimental support for the development of such plant-derived therapeutics targeting this disease.

## 1 Introduction

Obesity, as a globally prevalent metabolic disorder, is closely linked to the incidence and mortality of cardiovascular disease (CVD) (Piché et al., 2020; Tutor et al., 2023). It not only leads to metabolic disorders (such as dyslipidemia, insulin resistance) and chronic inflammation (Caballero, 2019; Purdy and Shatzel, 2021; Kawai et al., 2021), but also induces myocardial structural and functional remodeling through lipotoxicity-induced damage (Rosengren, 2021; Neeland et al., 2019). Excessive accumulation of free fatty acids in cardiomyocytes, as observed in obesity and metabolic disorders, triggers lipotoxicity and impairs mitochondrial homeostasis. Elevated free fatty acids and their toxic metabolites, including ceramides and diacylglycerol, induce mitochondrial structural damage, increase oxidative stress, disrupt mitochondrial fission–fusion dynamics, inhibit oxidative phosphorylation, and reduce ATP production. These lipotoxic alterations ultimately lead to mitochondrial dysfunction, cardiomyocyte injury, and impaired cardiac function, highlighting the critical role of mitochondrial damage in the pathogenesis of metabolic cardiomyopathy (Koliaki et al., 2019). Recent studies have identified that mitochondrial dynamics imbalance—excessive fission (e.g., Drp1, Fis1) and impaired fusion (e.g., Opa1, Mfn2)—is a key driver of obesity-associated cardiomyopathy (OCM) (Tong et al., 2023; Tokuyama and Yanagi, 2023). Since mitochondrial function is essential for maintaining cardiac contractility, as the heart relies heavily on mitochondrial ATP production, targeting the balance of mitochondrial fission and fusion represents a promising strategy in metabolic heart disease therapy (Feng et al., 2020; Hinton et al., 2024).

*Salvia miltiorrhiza* (Danshen), a traditional Chinese medicinal plant used for promoting blood circulation and removing blood stasis, contains liposoluble danshen ketones and water-soluble phenolic acids (Chen and Chen, 2017), which exhibit multiple effects such as anti-inflammatory, antioxidant, anti-fibrotic, and microcirculation -improving effects (Wu et al., 2024a; Li et al., 2018). Although most existing studies have focused on single components such as salvianolic acid B or tanshinone IIA, which can improve mitochondrial function by reducing oxidative stress, maintaining mitochondrial membrane potential, and regulating mitochondrial dynamics (Zhao et al., 2023a; Zhang et al., 2019), studies on the synergistic mechanisms of their combined use remain limited. Recent studies have emphasized that the multiple metabolites in traditional Chinese medicine formulas can produce an overall network regulatory effect through synergistic interactions that surpasses the effects of individual metabolites (Zhao et al., 2023b; Jiang et al., 2022), suggesting that the optimized formulation of multiple metabolites in *S. miltiorrhiza* may offer enhanced therapeutic benefits.

In our previous study, the optimal formulation composed of danshensu (DSS), salvianolic acid A (Sal-A), salvianolic acid B (Sal-B), and protocatechuic aldehyde (PAL), named SABP, was established using uniform design and orthogonal design (Shao et al., 2022), which has been validated for its anti-inflammatory and anti-vascular fibrosis effects in atherosclerosis and hypertension models (Wu et al., 2023a; Wu et al., 2023b). However, its role in ameliorating lipotoxic cardiac injury, particularly *via* mitochondrial dynamics regulation, remains unclear. *ApoE* ^−/−^ mice fed a high-fat diet exhibit pronounced dyslipidemia, systemic inflammation, and lipid accumulation, making them a well-established model for studying metabolic disorders and lipid-induced organ injury (Mao et al., 2023a). Therefore, this study aims to investigate the protective effects and underlying mechanisms of SABP in both palmitic acid-induced lipotoxic H9c2 cells and high-fat diet-induced *ApoE* ^−/−^ mouse models, with a specific focus on restoring mitochondrial dynamics. Our findings may provide new insights into multi-target interventions with medicinal plant formulations for obesity-related cardiomyopathy.

## 2 Materials and methods

### 2.1 Cell line and animals

The H9c2 rat cardiomyocyte cell line was kindly donated by the Key Laboratory of Integrated Liver and Kidney Disease Research of Chinese and Western Medicine, Hebei University of Traditional Chinese Medicine. 10 male C57BL/6 J mice (7 weeks old) and 20 age- and strain-matched *ApoE* ^−/−^ mice were purchased from Specific (Beijing) Biotechnology Co. (License No. SCXK (Beijing) 2019–0,010). All animals were housed in the Animal Experiment Center of Hebei University of Traditional Chinese Medicine under standard conditions (22° C ± 1 °C, 50% ± 2% relative humidity, and a 12-h light/dark cycle), with *ad libitum* access to food and water. High-fat diet (Beijing Botai Hongda Biotechnology Co., China, Cat. No. HD001, Batch No. 2024062201) was used in this study. The animal experiments were approved by the Animal Ethics Committee of Hebei University of Traditional Chinese Medicine (Ethics Approval No. DWLL202409012).

### 2.2 Drugs

**FIGURE 1:**
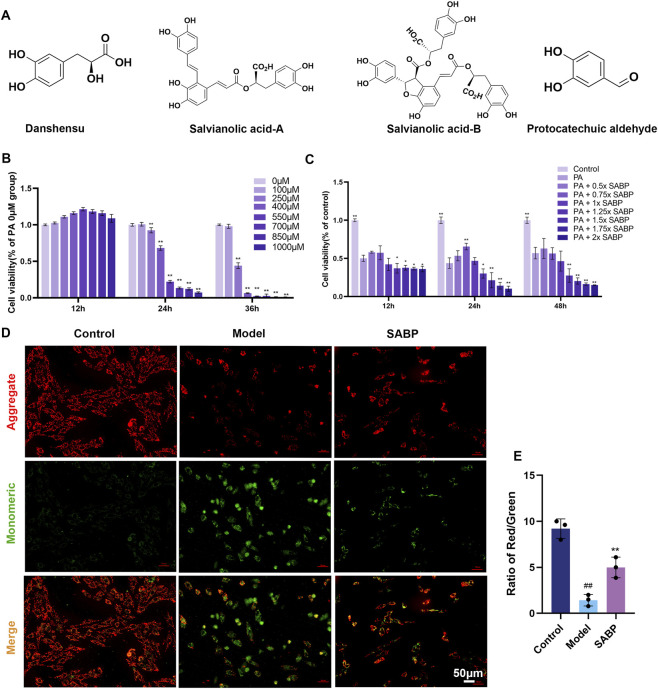
SABP increases H9c2 cell survival and improves mitochondrial function. **(A)** Chemical structures of the four metabolites in *Salvia miltiorrhiza* Bunge aqueous extract. **(B)** H9c2 cell viability following treatment with PA at different concentrations for 12, 24, 48 h tested by CCK-8 assay (*n* = 3). **(C)** H9c2 cell viability after treatment with 400 μM PA for 24 h and followed by different concentrations of SABP for 12, 24, and 48 h (*n* = 3). **(D,E)** Representative JC-1 fluorescence images and the Red/Green fluorescence ratio (n = 3 independent experiments; five to eight fields were analyzed per group per experiment). Scale bar: 50 μm. Data are expressed as mean ± SD. ^##^ *P* < 0.01 vs. Control group. ^**^ *P* < 0.01 vs. Model group.

The chemical structures of Danshensu (DSS), Salvianolic acid A (Sal-A), Salvianolic acid B (Sal-B), and Protocatechuic aldehyde (PAL) are shown in Figure 1. DSS, Sal-A, Sal-B, and PAL (Shanghai Jianglai Biotechnology Co., Ltd., China, Cat. Nos. J001459, A007573, A007574, P5585). Were all commercially obtained as chemically defined standards; therefore, no plant material authentication or voucher specimen was involved.

### 2.3 Reagents

The following reagents were used in this study: DMEM high-glucose medium (Gibco, United States of America, Cat. No. D0822), fetal bovine serum (Gibco, United States of America, Cat. No. F8687), penicillin-streptomycin solution (Solarbio, Beijing, China, Cat. No. P1400), palmitic acid (Sigma-Aldrich, Germany, Cat. No. P5585), and bovine serum albumin (BSA, Solarbio, Beijing, China, Cat. No. A8850) for cell culture and modeling.

Cell viability was assessed using the CCK-8 kit (Sheng’er Biotechnology, Shanghai, China), and mitochondrial membrane potential was measured with the JC-1 staining kit (Beyotime Biotechnology, China, Cat. No. C2003S). Serum levels of interleukin-6 (IL-6, Cat. No. SEKM-0007), interleukin-1β (IL-1β, Cat. No. SEKM-0002), tumor necrosis factor-α (TNF-α, Cat. No. SEKM-0034), and interleukin-10 (IL-10, Cat. No. SEKM-0010) were quantified using ELISA kits (Solarbio, Beijing, China). Lactate dehydrogenase (LDH, Cat. No. A020-one to two, Lot No. 20240,713) and creatine kinase-MB (CK-MB, Cat. No. H197-one to one, Lot No. 20240,809) detection kits were obtained from Nanjing Jiancheng Bioengineering Institute (China).

Histological analysis was performed using hematoxylin-eosin (HE) staining kit (Solarbio, Cat. No. G1120) and Masson’s trichrome staining kit (Solarbio, Cat. No. G1340). For transmission electron microscopy, fixative solution was purchased from Servicebio (Wuhan, China, Cat. No. G1102).

Western blotting was performed using primary antibodies against Drp1 (12957-1-AP), Fis1 (10956-1-AP), Opa1 (27733-1-AP), Mfn2 (12186-1-AP), cleaved caspase-3 (25128-1-AP), and caspase-3 (19677-1-AP), along with goat anti-rabbit secondary antibody (20,000,135), all from San Ying Biotechnology (Wuhan, China).

### 2.4 Instruments

Cell culture incubator (Thermo Fisher Scientific, United States of America), multifunctional microplate reader (PerkinElmer, Germany), fluorescence microscope (Nikon, Japan), automatic biochemical analyzer (LABOSPECT 006, Hitachi, Japan), small animal ultrasound imaging system (VisualSonics, Canada), micro-computed tomography (Micro-CT) system (Pingsheng Technology, China), transmission electron microscope (HT7700, Hitachi, Japan), and electrophoresis/electroblotting systems (Bio-Rad, United States of America) were used in this study.

### 2.5 Preparation of experimental reagents

The optimal ratio and concentrations of SABP were obtained from our previous study (Shao et al., 2022), which was optimized by uniform design and orthogonal design, and identified the most effective combination with synergistic biological activity. The specific ratio of DSS: Sal-A: Sal-B: PAL was 150: 7: 300: 500, with the corresponding concentrations of 1.5 × 10^−4^ mol/L, 7 × 10^−6^ mol/L, 3 × 10^−4^ mol/L, and 5 × 10^−4^ mol/L, respectively. The animals were administered with the dose of DSS, 5 mg/kg; Sal-A, 0.233 mg/kg; Sal-B, 10 mg/kg; and PAL, 17 mg/kg.

Palmitic acid (PA) solution was prepared as follows: defatted bovine serum albumin (BSA) was fully dissolved in phosphate-buffered saline (PBS) and PA was dissolved in a sodium hydroxide solution, then the two solutions were rapidly mixed to yield a stable brown-yellow PA-BSA complex solution. The final concentrations of PA,NAOH and BSA in the culture medium were 400 μM, 1 mM and 0.4%, respectively.

### 2.6 Cell culture and grouping

H9c2 cells were cultured in high-glucose DMEM supplemented with 10% fetal bovine serum and 1% penicillin-streptomycin, and maintained in a humidified incubator at 37 °C with 5% CO_2_. Cells in the logarithmic growth phase were used for subsequent experiments. Cells were divided into three groups: control group (cultured under standard conditions without treatment), model group (treated with PA for 24 h), and SABP group (co-treated with PA and SABP at the optimized concentration for 24 h). In this study, SABP was prepared from chemically defined pure metabolites (DSS, Sal-A, Sal-B, and PAL), allowing precise control of concentration and composition. Therefore, unlike traditional serum pharmacology approaches, SABP was directly applied to cells to evaluate its direct biological effects and underlying mechanisms. This design was intended to simulate lipotoxic stress and evaluate the direct protective effects of SABP on cardiomyocytes.

### 2.7 Preparation of the palmitic acid-induced myocardial lipotoxicity model

H9c2 cells were seeded into 96-well plates at a density of 1 × 10^4^ cells per well and cultured at 37 °C for 24 h. Then, eight PA concentrations (0, 100, 250, 400, 550, 700, 850 and 1,000 μM) were used, with six replicates per concentration group. Cell viability was assessed at 12, 24, and 48 h after PA exposure using the CCK-8 assay. Specifically, 10 μL of CCK-8 solution was added to each well for 1 h, and absorbance was measured at 450 nm using a microplate reader. The average absorbance of the six wells was used as the viability value for each group. This concentration-time optimization ensured a stable and reproducible lipotoxicity model for subsequent intervention experiments.

### 2.8 Effect of SABP on H9c2 cell viability measured by CCK-8 assay

H9c2 cells were seeded into 96-well plates at a density of 1 × 10^4^ cells/well and cultured at 37 °C for 24 h. Lipotoxicity was induced by treating the cells with 400 μM PA for 24 h. After modeling, the culture medium was replaced.

Cells were then divided into the following groups: (1) normal control group (no PA or SABP treatment), (2) PA model group (treated with PA only), and (3) SABP treatment groups (treated with PA and varying concentrations of SABP: 0.5×, 0.75×, 1×, 1.25×, 1.5×, 1.75×, and 2×).

After incubation for 12, 24, and 48 h, 10 μL of CCK-8 reagent was added to each well. Cells were incubated with CCK-8 reagent for 1 h before measurement. Absorbance was measured at 450 nm, and the results were reported as the mean absorbance from six replicate wells per group. Dose-response analysis was performed to identify the optimal effective concentration of SABP for mechanistic studies.

### 2.9 JC-1 staining for mitochondrial membrane potential

Mitochondrial membrane potential (ΔΨm) was assessed using the JC-1 fluorescent probe (Beyotime, Cat. No. C2003S). In healthy mitochondria with high membrane potential, JC-1 aggregates and emits red fluorescence, while in depolarized mitochondria, it remains in monomeric form and emits green fluorescence.

H9c2 cells (4 × 10^4^ cells/well) were seeded in 12-well plates. After model induction with or without SABP, JC-1 working solution was added, and cells were incubated at 37 °C for 20 min in the dark. After washing with PBS, 1 mL of complete culture medium was added to each well, and fluorescence images were captured using a fluorescence microscope.

### 2.10 Animal grouping and treatment

After 1 week of acclimatization, ten C57BL/6 J mice and twenty *ApoE* ^−/−^ mice were used in the study. C57BL/6 J mice were fed a standard diet and served as the control group; *ApoE* ^−/−^ mice were given a high-fat diet for eight consecutive weeks to induce the model and were then randomly divided into two groups: the model group and the SABP treatment group. Following successful model establishment, mice in the SABP group received daily oral gavage of the SABP mixture for 8 weeks, while mice in the control and model groups received equal volumes of normal saline. This model was selected to mimic obesity-associated metabolic disturbances and systemic lipid overload, which are key contributors to cardiac lipotoxicity.

### 2.11 Echocardiography of small animal

Echocardiography was performed to evaluate cardiac function in each group of mice. Mice were anesthetized with isoflurane inhalation, and the chest was deflowered and placed in a supine position on the ultrasound platform after chest hair removal. The probe was placed perpendicular to the left chest wall at an angle of 25° to the sternum to visualize the long axis of the left ventricle from the mitral orifice to the apex, and then the probe was rotated by 90°to visualize the short axis view perpendicular to the long axis. Left ventricular end-diastolic diameter (LVEDD) and left ventricular end-systolic diameter (LVESD) were measured in M-mode. Left ventricular ejection fraction (LVEF) and fractional shortening (LVFS) were calculated using the accompanying analysis software. Each parameter was measured three times per mouse, and the mean value was used for statistical analysis.

### 2.12 Micro-CT examination of small animals

Micro-computed tomography (Micro-CT) was performed in each group of mice. Mice were anesthetized in an isoflurane-containing anesthesia chamber and positioned within the Micro-CT scanner. Cruiser software (version 2.0.11.0) was used, with the scanning parameters as follows: mouse visceral scan mode, tube voltage 60 kV, tube current 150 μA, frame rate 20 fps, Rebin mode 1 × 1, detector-to-source distance (DSD) 410 mm, and maximum transverse field of view (FOV) 50 mm.

Image reconstruction was performed using Recon Daemon software (version 2.0.11.0), and the 3D reconstruction and analysis were conducted using Avatar3 software (version 2.0.11.0). The CT 3D reconstruction algorithm was iterative, with a resolution of 1,024 × 1,024 pixels (1 K × 1 K), a field of view of 30 mm, slice thickness of 0.03 mm, pixel size of 0.03 mm, and three iterations. Fat tissue in anatomically identical regions was quantitatively analyzed. The analysis included measurements of total body volume and fat volume for each mouse.

### 2.13 Heart mass index and heart-tibia ratio

After anesthesia, the mice were euthanized and their hearts were excised. After removing surrounding fat and major blood vessels, the hearts were weighed. Simultaneously, the fasting body weight and the length of the right tibia were measured. The heart weight-to-body weight ratio (heart mass index) and the heart weight-to-tibia length ratio (heart-tibia index) were calculated for each group.

### 2.14 Biochemical analysis

After mice were anesthetized by isoflurane inhalation, venous blood was collected from the retro-orbital sinus. Blood samples were left at room temperature for 1 h and then centrifuged at 3,000 rpm for 15 min to obtain serum.

Serum lipid levels, including total cholesterol (TC), triglycerides (TG), low-density lipoprotein cholesterol (LDL-C), and high-density lipoprotein cholesterol (HDL-C), were measured using an automated biochemical analyzer. Inflammatory cytokines (IL-6, IL-1β, TNF-α, and IL-10) and cardiac injury markers (lactate dehydrogenase [LDH] and creatine kinase-MB [CK-MB]) were quantified using commercial ELISA kits according to the manufacturer’s instructions. Absorbance was measured at 450 nm, and concentrations were calculated based on standard curves.

### 2.15 Histological and ultrastructural analysis of cardiac tissue

For histological evaluation, apical heart tissue was fixed, dehydrated, embedded in paraffin, and sectioned. Hematoxylin-eosin (HE) staining and Masson’s trichrome staining were performed following standard protocols. Sections were observed under a light microscope and imaged for histopathological analysis.

For ultrastructural observation by transmission electron microscopy (TEM), samples from the left ventricular myocardium were cut into ∼1 mm^3^ cubes and fixed in 2.5% glutaraldehyde at 4° Covernight. The tissue was then rinsed in phosphate-buffered saline, post-fixed in 1% osmium tetroxide, dehydrated through a graded ethanol and acetone series, embedded in resin, and ultrathin sections (∼70 nm) were obtained.

Sections were stained with 2% aqueous uranyl acetate and lead citrate, and examined under a TEM. Mitochondrial morphology and myocardial ultrastructure were assessed. Quantitative analysis of mitochondrial number and average area per unit field was conducted using ImageJ software (Feng et al., 2020).

### 2.16 Western blot analysis

Western blotting was performed to assess the expression of proteins related to mitochondrial dynamics and apoptosis. Total protein was extracted from myocardial tissues and H9c2 cells using RIPA lysis buffer, and protein concentrations were determined using a bicinchoninic acid assay (BCA).

Equal amounts of protein (10–20 μg) were separated by SDS-polyacrylamide gel electrophoresis (SDS-PAGE) and transferred onto polyvinylidene fluoride (PVDF) membranes. Membranes were blocked with 5% non-fat milk in TBST for 1 h at room temperature, then incubated overnight at 4 °C with primary antibodies against Drp1, Fis1, Opa1, Mfn2, cleaved caspase-3, and caspase-3, and followed by incubation with horseradish peroxidase-conjugated goat anti-rabbit secondary antibody for 2 h at room temperature. Protein bands were visualized using enhanced chemiluminescence (ECL) reagents and imaged with a chemiluminescence imaging system. Band intensities were quantified using ImageJ software, and the relative expression levels were normalized to β-actin.

### 2.17 Statistical analysis

GraphPad Prism 9.5 (GraphPad Software, United States of America) was used for statistical analysis and graphical representation. All data were presented as mean ± standard deviation (SD), and one-way analysis of variance (ANOVA) was used for multi-group comparison. A *p-value* < 0.05 was considered statistically significant with *p* < 0.01 indicating highly significant.

## 3 Results

### 3.1 SABP increases H9c2 cell survival and improves mitochondrial function

To evaluate the protective effect of SABP on H9c2 cells under lipotoxic conditions, this study firstly established a model of myocardial lipotoxicity induced by palmitic acid (PA) and the optimal modeling and treatment conditions were determined. CCK-8 assay results showed that treatment with 400 μM PA for 24 h reduced H9c2 cell viability to approximately 60%, therefore, 400 μM PA for 24 h was selected for the subsequent modeling condition (Figure 1B).

The effect of different concentrations of SABP on cell viability was further investigated, and the results showed that the highest cell viability was observed with 0.75 × SABP treatment for 24 h, indicating a potential protective effect, and thus this concentration and time point were used in further experiments (Figure 1C).

Mitochondrial membrane potential was assessed using JC-1 staining. The red/green fluorescence ratio in the model group was significantly decreased (*P* < 0.01) compared with that of the control group, suggesting that mitochondrial membrane potential was significantly reduced; whereas, the ratio was significantly increased (*P* < 0.01) after the intervention of SABP, which indicated that SABP could effectively improve the mitochondrial membrane potential and attenuate PA-induced mitochondrial dysfunction (Figures 1D,E).

### 3.2 SABP regulates mitochondrial dynamics and apoptotic protein expression in H9c2 cells

To further investigate the regulation of mitochondrial function and apoptosis by SABP at the cellular level, the expression of mitochondrial dynamics and apoptosis-related proteins were detected.

**FIGURE 2:**
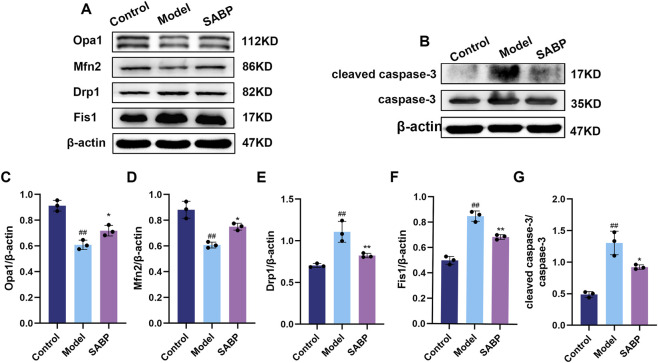
SABP regulates mitochondrial dynamics and apoptotic protein expression in H9c2 cells. **(A–G)** Expression levels of Opa1, Mfn2, Drp1, Fis1 **(A,C–F)** and caspase-3, cleaved caspase-3 **(B,G)** in H9c2 cells determined by Western blot (*n* = 3). Data are expressed as mean ± SD. ^##^ *P* < 0.01 vs. Control group. ^*^ *P* < 0.05, ^**^ *P* < 0.01 vs. Model group.

Compared with the control group, the expression of mitochondrial split proteins Drp1 and Fis1 were upregulated and the expression of fusion proteins Opa1 and Mfn2 were downregulated in H9c2 cells of the model group (*P* < 0.01), suggesting an imbalance of mitochondrial dynamics under lipotoxicity; SABP treatment reversed the above abnormality, significantly down-regulating the expression of Drp1 and Fis1 and up-regulating the expression of Opa1, Mfn2 (*P* < 0.05) (Figures 2A,C–F), suggesting that it helps to restore mitochondrial kinetic balance.

Moreover, the cleaved caspase-3/caspase-3 ratio was significantly elevated in the model group (*P* < 0.01), indicating enhanced apoptosis. SABP treatment effectively reduced the ratio (*P* < 0.05), suggesting that SABP effectively inhibited PA-induced apoptosis in cardiomyocytes *in vitro* (Figures 2B,G).

### 3.3 SABP improves cardiac function and reduces fat accumulation in high-fat diet induced mice

**FIGURE 3:**
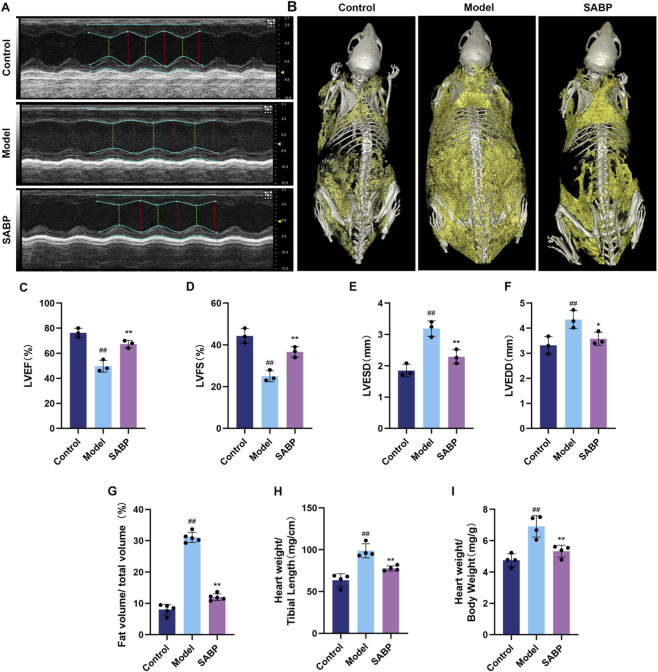
SABP improves cardiac function and reduces fat accumulation in high-fat-diet induced mice. **(A,C–F)** Representative images of echocardiography and measurements of LVEF, LVFS, LVESD and LVEDD (*n* = 3). **(B,G)** Representative images of Micro-CT and fat volume-to-total volume ratio in mice (*n* = 3). **(H)** Ratio of heart weight to tibia length (*n* = 3). **(I)** Heart weight to body weight ratio (*n* = 3). Data are expressed as mean ± SD. ^##^ *P* < 0.01 vs. Control group. ^*^ *P* < 0.05, ^**^ *P* < 0.01 vs. Model group.

To evaluate the effects of SABP on cardiac function and lipid metabolism in high-fat-diet-induced mice, several *in vivo* assays were performed after treatment. Echocardiographic results showed that compared with the control group, mice in the model group exhibited significantly reduced left ventricular ejection fraction (LVEF) and fractional shortening (LVFS), along with increased left ventricular end-systolic diameter (LVESD) and end-diastolic diameter (LVEDD) (*P* < 0.01). SABP intervention significantly increased LVEF and LVFS (*P* < 0.01), and decreased LVESD and LVEDD (*P* < 0.05), indicating enhanced cardiac systolic function (Figures 3A,C–F).

Micro-CT 3D reconstruction results showed a significantly higher fat volume-to-total volume ratio in the model group compared with the control group (*P <* 0.01). This ratio was significantly decreased by SABP treatment (*P* < 0.01), indicating a lipid-lowering effect (Figures 3B,G). In addition, both the heart-to-tibia ratio and heart weight index were significantly elevated in model group (*P* < 0.01), while SABP significantly reduced these two parameters (*P* < 0.01) (Figures 3H,I), further supporting the effect of SABP in improving cardiac fat accumulation.

### 3.4 SABP ameliorates dyslipidemia and inflammatory response and alleviates myocardial injury in high-fat diet-induced mice

**FIGURE 4:**
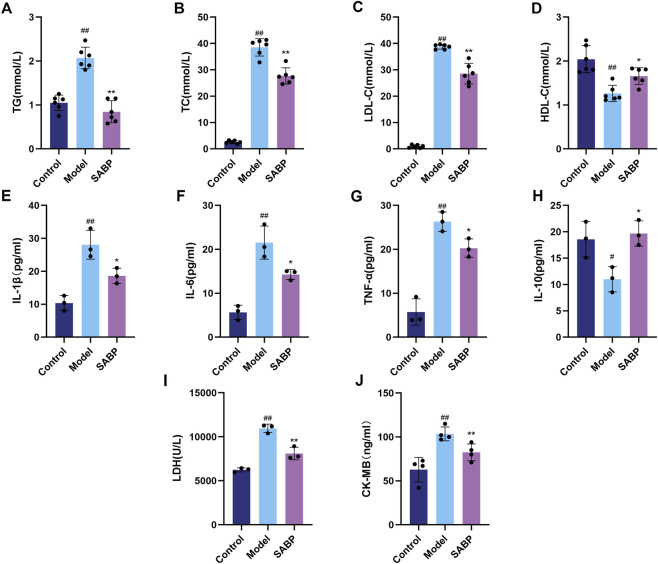
SABP ameliorates dyslipidemia and inflammatory response and alleviates myocardial injury in high-fat diet-induced mice. **(A–D)** Serum lipid and TG, TC, LDL-C, HDL-C levels in mice. **(E–H)** Levels of IL-1β, IL-6, TNF-α and IL-10 in serum. **(I,J)** Levels of LDH and CK-MB in serum. Data are expressed as mean ± SD. ^#^ *P* < 0.05, ^##^ *P* < 0.01 vs. Control group. ^*^ *P* < 0.05, ^**^ *P* < 0.01 vs. Model group.

To further assess the role of SABP in metabolic regulation and cardioprotection, serum levels of lipids, inflammatory factors and cardiac enzymes were measured. Compared with the control group, the serum levels of triglyceride (TG), total cholesterol (TC) and low-density lipoprotein cholesterol (LDL-C) in the model group were significantly elevated, along with the level of high-density lipoprotein cholesterol (HDL-C) was significantly decreased (*P* < 0.01); SABP treatment significantly reduced TG, TC, and LDL-C levels (*P* < 0.01) and modestly increased HDL-C levels (*P* < 0.05) (Figures 4A–D), indicating an improvement in lipid metabolism.

Pro-inflammatory cytokines, including IL-1β, IL-6 and TNF-α were significantly elevated in the model group (*P* < 0.01), while the level of anti-inflammatory factor IL-10 was decreased (*P* < 0.05). SABP intervention significantly downregulated IL-1β, IL-6, and TNF-α levels (*P* < 0.05) and upregulated IL-10 level (*P* < 0.05) (Figures 4E–H), suggesting an anti-inflammatory effect.

Furthermore, ELISA results showed that the serum levels of lactate dehydrogenase (LDH) and creatine kinase isoenzyme MB (CK-MB), markers of myocardial injury, were significantly elevated in the model group (*P* < 0.01), both of which were significantly reduced following SABP treatment (*P* < 0.01) (Figures 4I,J), suggesting that SABP alleviates myocardial injury.

### 3.5 SABP alleviates myocardial tissue damage and mitochondrial ultrastructural abnormalities in high-fat diet-induced mice

To investigate the effects of SABP on high-fat diet-induced myocardial structural damage and mitochondrial morphological changes, histological and electron microscopy analyses were performed.

**FIGURE 5:**
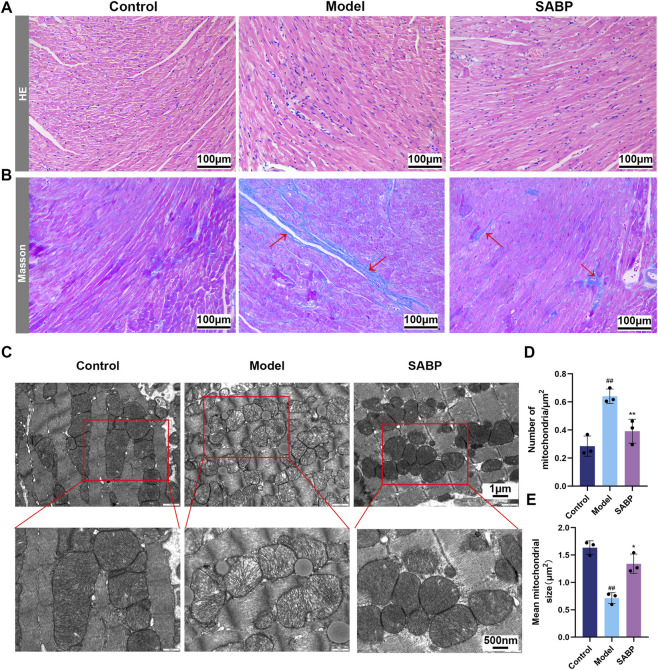
SABP alleviates myocardial tissue damage and mitochondrial ultrastructural abnormalities in high-fat-induced mice. **(A,B)** Representative H&E **(A)** and Masson’s trichrome staining **(B)** of the left ventricular apex. Scale bar: 50 μm. **(C)** Representative TEM images of damaged mitochondria. Scale bars: top row, 2 μm; bottom row, 1 μm. **(D)** Quantitative analysis of the number of mitochondria per μm^2^ (*n* = 3). **(E)** Quantitative analysis of the mean mitochondrial size (*n* = 3). Data are expressed as mean ± SD. ^##^ *P* < 0.01 vs. Control group. ^*^ *P* < 0.05, ^**^ *P* < 0.01 vs. Model group.

Hematoxylin-eosin (HE) staining showed that myocardial tissue in the control group exhibited intact structure, orderly arranged cardiomyocytes, and no obvious inflammatory infiltration. In contrast, the model group showed significant myocardial swelling, fiber breakage, disorganized cellular structure, and widespread inflammatory cell infiltration. SABP treatment alleviated myocardial swelling and inflammation, and improved cellular organization (Figure 5A).

Masson’s trichrome staining revealed no evident fibrosis in the control group, while the model group showed marked collagen fiber deposition. SABP treatment significantly reduced collagen deposition and alleviate myocardial fibrosis (Figure 5B).

Transmission electron microscopy (TEM) further demonstrated that mitochondria in the control group had intactmorphology and clear cristae. In the model group, there was extensive lipid droplet accumulation, mitochondrial swelling, cristae disruption, vacuolization, increased mitochondrial number (*P* < 0.01), and reduced average mitochondrial area (*P* < 0.01). SABP treatment restored mitochondrial integrity, decreased vacuolization, and improved mitochondrial ultrastructural organization, as evidenced by a reduced mitochondrial count (*P* < 0.01) and increased average area (*P* < 0.05) (Figures 5C–E).

### 3.6 SABP regulates mitochondria-related protein expression and inhibits myocardial apoptosis to alleviate high-fat diet-induced myocardial injury in mice

To verify whether SABP also ameliorates myocardial injury *in vivo* by regulating mitochondrial dynamics and apoptosis-related pathways, Western blotting was performed on myocardial tissue samples.

**FIGURE 6:**
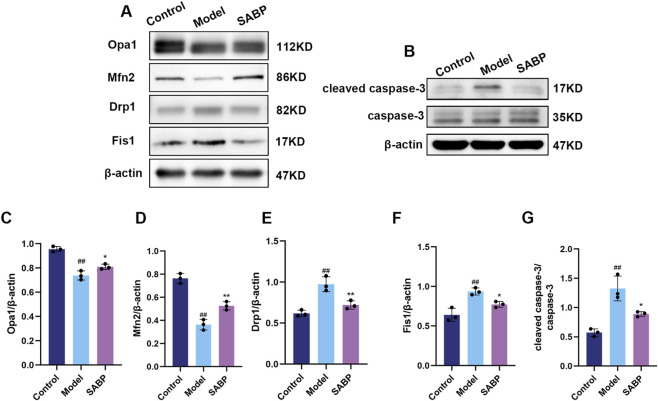
SABP regulates mitochondria-related protein expression and inhibits myocardial apoptosis to alleviate high-fat-induced myocardial injury in mice. **(A,C–F)** Protein expression of Opa1, Mfn2, Drp1, Fis1 in cardiac tissue (*n* = 3). **(B,G)** Apoptosis related protein expression detected by Western blot and the ratio of cleaved-caspase 3/caspase-3 (*n* = 3). Data are expressed as mean ± SD. ^##^ *P* < 0.01 vs. Control group. ^*^ *P* < 0.05, ^**^ *P* < 0.01 vs. Model group.

Animal-level Western blot analysis showed that the expression of Drp1 and Fis1 was upregulated and the expression of Opa1 and Mfn2 was decreased in the myocardial tissues of mice in the model group compared with that of the normal group (*P* < 0.01), confirming mitochondrial dysfunction under high-fat dietary conditions. SABP administration effectively restored their expression levels and improved the balance of mitochondrial fusion and division (*P* < 0.05) (Figures 6A,C–F), indicating improved mitochondrial dynamics *in vivo*.

Furthermore, the cleaved caspase-3/caspase-3 ratio in myocardial tissues of the model group was also significantly elevated (*P* < 0.01), and significantly decreased following SABP treatment (*P* < 0.05) (Figures 6B,G), suggesting that SABP attenuates lipotoxicity-induced myocardial apoptosis and contributes to myocardial protection *in vivo*.

## 4 Discussion

Obesity, characterized by excessive fat accumulation, is an important risk factor for various cardiovascular diseases, including atherosclerosis and heart failure (Piché et al., 2020; Tutor et al., 2023; Mao et al., 2023a). Adipose tissue serves not only as an energy reservoir but also as the largest endocrine organ in the body, which can influence systemic metabolism by secreting a variety of cytokines (Iacobellis, 2023; Zwick et al., 2018; Ren et al., 2021). In obesity, dysfunctional adipose tissue leads to endocrine disorders, hyperglycemia, dyslipidemia, and insulin resistance (Mao et al., 2023b; Kajikawa and Higashi, 2022; Carobbio et al., 2024), induces hypoxia, inflammatory response, and oxidative stress, which further aggravate endoplasmic reticulum stress and mitochondrial dysfunction. Consequently, these pathophysiological changes accelerate apoptosis, and ultimately contribute to metabolic disorders and structural remodeling of the myocardium (Landini et al., 2016; Marcelin et al., 2022; Yazıcı et al., 2017; Nosalski and Guzik, 2017).

In this study, we simulated the state of obesity-associated myocardial injury through a high-fat diet-induced mouse model, and found that SABP intervention effectively improved dyslipidemia (decreased TC, TG, LDL-C, increased HDL-C), attenuated adipose tissue expansion as confirmed by Micro-CT, reduced inflammatory responses (lower levels of TNF-α, IL-6, IL-1β; higher IL-10 expression), alleviated myocardial injury (reduced serum LDH, CK-MB; improved histopathology and reduced fibrosis) and significantly improved cardiac function (increased LVEF, LVFS), suggesting that SABP has a comprehensive intervention effect on obesity-associated myocardial injury through modulation of lipid metabolism and inflammation.

Heart is a highly energy-dependent organ, with its metabolism primarily relying on mitochondrial β-oxidation of fatty acids under physiological conditions (Da Dalt et al., 2023; Bodenstab et al., 2024). In obesity, elevated plasma free fatty acid levels shift substrate preference in cardiomyocytes toward excessive fatty acid uptake and oxidation, leading to the accumulation of toxic lipid intermediates, a process known as lipotoxicity (Lopaschuk et al., 2007). These intermediates can induce excessive mitochondrial oxidative stress, decreased membrane potential and dysfunction, ultimately leading to cardiomyocyte apoptosis and impaired cardiac function (Ussher and Aasum, 2024; Sletten et al., 2018; Wu et al., 2024b; Chaurasia and Summers, 2021). In this study, we found that SABP significantly increased cell viability and maintained mitochondrial membrane potential stability in the palmitic acid-induced lipotoxicity model of H9c2 cells, indicating that SABP exerts mitochondrial protective effects.

Further mechanistic investigations showed that SABP prevents lipotoxicity-induced injury by restoring mitochondrial dynamic homeostasis, which is tightly regulated by fission and fusion, which are essential for maintaining mitochondrial function and cellular energy balance (Jin et al., 2021; Tong et al., 2023; Feng et al., 2020; Tokuyama and Yanagi, 2023). Mitochondrial fission is primarily mediated by the recruitment of dynamin-related protein 1 (Drp1) to the outer mitochondrial membrane through adaptor proteins such as Fis1. Fusion, on the other hand, is mediated by optic atrophy protein 1 (Opa1) for inner membrane fusion and mitofusins (Mfn1/2) for outer membrane fusion. Under physiological conditions, mitochondrial fission facilitates the removal of damaged mitochondria and improves mitochondrial quality, but excessive fission causes an increase in the number of mitochondria and a decrease in their size, leading to mitochondrial fragmentation and impaired function (Feng et al., 2020; Tokuyama and Yanagi, 2023). Increasing evidence suggests that an imbalance between mitochondrial fission and fusion is a central mechanism in obesity-associated cardiomyopathy (Chen et al., 2023). In particular, Drp1 overactivation and Opa1/Mfn2 downregulation have important roles in mediating cardiac lipotoxicity and dysfunction (Tong et al., 2023).

Our findings demonstrate that SABP intervention significantly downregulates the expression of pro-mitotic proteins Drp1 and Fis1, while upregulates the expression of pro-fusion proteins Opa1 and Mfn2 in H9c2 cells and mouse heart tissue exposed to lipotoxic stress (PA or HFD), as evidenced by improvements in mitochondrial ultrastructural damage. These ultrastructural observations were consistent with the changes in mitochondrial dynamics-related protein expression. However, it should be noted that TEM-based quantification primarily reflects morphological alterations and does not directly capture dynamic processes; therefore, these findings should be interpreted in combination with molecular markers of mitochondrial dynamics. Moreover, SABP treatment markedly reduced the cleaved caspase-3/caspase-3 ratio in both *in vitro* and *in vivo* models, indicating effective inhibition of lipotoxicity-induced apoptosis. These results suggest that the protective effect of SABP may be partially achieved by regulating mitochondrial dynamics, thereby attenuating mitochondrial-dependent apoptotic pathways. This is consistent with recent studies highlighting the role of mitochondrial fission/fusion imbalance and apoptotic signaling in the progression of heart failure (Liu et al., 2024; Yan et al., 2024). Although the present study demonstrated that SABP significantly reduced lipid accumulation at the systemic level, the precise molecular mechanisms underlying this effect remain to be fully elucidated. Given the central role of mitochondria in fatty acid β-oxidation, and the observed restoration of mitochondrial dynamics in this study, it is plausible that SABP may enhance lipid utilization by improving mitochondrial metabolic function. Further studies involving lipid quantification and metabolic flux analysis are required to validate this hypothesis. More advanced approaches such as live-cell imaging or confocal microscopy would further strengthen the evaluation of mitochondrial dynamics.

It is worth noting that, unlike previous studies focusing on single metabolite of *S. miltiorrhiza*, the present study systematically investigated an optimized multi-metabolites formulation (SABP), demonstrating its integrated regulatory effects on lipid metabolism, inflammation, and mitochondrial dynamics. Importantly, this study highlights mitochondrial dynamics as a central mechanistic axis linking metabolic disorder to cardiomyocyte injury, and reveals that SABP exerts protective effects by restoring the balance between mitochondrial fission and fusion. In addition, a multi-scale evaluation system was established, integrating cellular experiments, animal models, and ultrastructural analysis, which provides more comprehensive evidence compared to conventional single-level studies. These findings underscore the advantage of multi-component synergistic intervention and network regulation in medicinal plant (Wu et al., 2020; Wang et al., 2024), offering new insights into therapeutic strategies for metabolic cardiomyopathy.

Nonetheless, this study has several limitations. First, although mitochondrial morphology was examined by TEM, advanced methods such as live-cell imaging or confocal microscopy were not used to further characterize mitochondrial dynamics. Second, lipid staining and quantitative lipid analysis were not performed; thus, the precise mechanism by which SABP regulates lipid storage and fatty acid oxidation remains unclear. Third, the lack of dose–response groups and positive controls limits the evaluation of therapeutic efficacy. Fourth, target-specific validation (e.g., Drp1 silencing or inhibition) was not conducted to confirm causality. In addition, the absence of control groups (SABP-only *in vitro*, wild-type HFD, or ApoE^−/−^ normal diet) restricts further distinction between genetic and dietary effects. Future studies will address these limitations to clarify the mechanisms and translational potential of SABP. Despite these limitations, our multi-level cellular, molecular, and ultrastructural evidence supports that SABP protects against lipotoxic cardiac injury and may represent a promising multi-target agent for obesity-related cardiomyopathy.

## 5 Conclusion

This study systematically revealed the formulation of water-soluble metabolites of *S. miltiorrhiza* (DSS, Sal-A, Sal-B, PAL) exerts cardioprotective effects against lipotoxicity-induced injury at both cellular and animal levels. SABP improved dyslipidemia and inflammation, inhibited cardiomyocyte apoptosis, and most notably, restored mitochondrial dynamic balance. These effects highlight the synergistic advantages of multi-metabolite formulations over single component in traditional research.

In addition, a multi-scale evaluation system from whole-body to subcellular levels was established, by integrating Micro-CT, dynamic fat monitoring, and ultrastructural analysis *via* transmission electron microscopy, clarifying the mechanisms of SABP in regulating the metabolism-inflammation-mitochondria- apoptosis cascade.

Altogether, our findings provide a theoretical foundation for the application of medicinal plant formulas in the prevention and treatment of obesity-related cardiomyopathy and support their translational potential in addressing cardiovascular metabolic diseases.

## Data Availability

The original contributions presented in the study are included in the article/supplementary material, further inquiries can be directed to the corresponding authors.

## Glossary

BCA: bicinchoninic acid assay
BSA: bovine serum albumin
CK-MB: Creatine Kinase–Myocardial Band
CVD: cardiovascular disease
Drp1: Dynamin-related protein 1
DSD: detector-to-source distance
DSS: danshensu
ECL: enhanced chemiluminescence
Fis1: Mitochondrial fission 1 protein
HDL-C: high-density lipoprotein cholesterol
HE: hematoxylin-eosin
HFD: high-fat diet
IL-10: interleukin-10
IL-1β: interleukin-1β
IL-6: interleukin-6
LDH: Lactate dehydrogenase
LDL-C: low-density lipoprotein cholesterol
LVEDD: Left ventricular end-diastolic diameter
LVEF: Left ventricular ejection fraction
LVESD: left ventricular end-systolic diameter
LVFS: Left ventricular fractional shortening
Mfn2: Mitofusin 2
OCM: obesity-associated cardiomyopathy
Opa1: Optic atrophy 1
PA: Palmitic acid
PAL: protocatechuic aldehyde
PBS: phosphate-buffered saline
PVDF: polyvinylidene fluoride
Sal-A: salvianolic acid A
Sal-B: salvianolic acid B
TC: total cholesterol
TEM: transmission electron microscopy
TG: triglycerides
TNF-α: tumor necrosis factor-α
